# Cardiac Ion Channelopathies and the Sudden Infant Death Syndrome

**DOI:** 10.5402/2012/846171

**Published:** 2012-12-05

**Authors:** Ronald Wilders

**Affiliations:** Department of Anatomy, Embryology and Physiology, Heart Failure Research Center, Academic Medical Center, University of Amsterdam, P.O. Box 22700, 1100 DE Amsterdam, The Netherlands

## Abstract

The sudden infant death syndrome (SIDS) causes the sudden death of an apparently healthy infant, which remains unexplained despite a thorough investigation, including the performance of a complete autopsy. The triple risk model for the pathogenesis of SIDS points to the coincidence of a vulnerable infant, a critical developmental period, and an exogenous stressor. Primary electrical diseases of the heart, which may cause lethal arrhythmias as a result of dysfunctioning cardiac ion channels (“cardiac ion channelopathies”) and are not detectable during a standard postmortem examination, may create the vulnerable infant and thus contribute to SIDS. Evidence comes from clinical correlations between the long QT syndrome and SIDS as well as genetic analyses in cohorts of SIDS victims (“molecular autopsy”), which have revealed a large number of mutations in ion channel-related genes linked to inheritable arrhythmogenic syndromes, in particular the long QT syndrome, the short QT syndrome, the Brugada syndrome, and catecholaminergic polymorphic ventricular tachycardia. Combining data from population-based cohort studies, it can be concluded that at least one out of five SIDS victims carries a mutation in a cardiac ion channel-related gene and that the majority of these mutations are of a known malignant phenotype.

## 1. Sudden Infant Death Syndrome

Unlike other syndromes, the diagnosis of the sudden infant death syndrome (SIDS) is one of exclusion. Another particular feature of the syndrome is that all of its carriers are dead at the time of diagnosis. Not surprisingly, SIDS does not have a clear-cut pathophysiology. A large number of pathophysiological mechanisms have been suggested and investigated, including uncontrolled inflammatory responses, serotonergic abnormalities, and metabolic disorders. Presumably, SIDS is a multifactorial disorder, with several intrinsic and extrinsic factors resulting in or predisposing to its development, as proposed in the “triple risk model.” In the present section, I will present and discuss the various definitions of SIDS, its prevalence, common risk factors, the triple risk model, and some noncardiac genetic predispositions.

### 1.1. Definition

The sudden and unexplained death of an apparently healthy infant is a tragic event. Typically, the infant is routinely put to sleep and found dead when one of the parents takes a look, which may be the next morning after having put the infant to sleep for the night or only minutes after having put the infant to sleep for a nap in the morning or afternoon. For such death, commonly known as “crib death” or “cot death,” the term “sudden infant death syndrome” may be used, provided that certain definitional conditions are met. The first definition of SIDS was proposed at the Second International Conference on Causes of Sudden Death in Infants, which was held in Seattle in 1969. This definition, which is known as the “Seattle definition,” states that SIDS is “the sudden death of any infant or young child which is unexpected by history and in which a thorough postmortem examination fails to demonstrate an adequate cause of death” [1]. This definition was revised by an expert panel convened by the National Institute of Child Health and Human Development (NICHD) in 1989. This revised definition is known as the “NICHD definition” and states that SIDS is “the sudden death of an infant under one year of age which remains unexplained after a thorough case investigation, including performance of a complete autopsy, examination of the death scene, and review of the clinical history” [2]. It thus limits the age of the SIDS victim to <1 year and specifies that the thorough examination should include complete autopsy and review of the death scene and clinical history. Of note, in an accompanying statement the expert panel emphasized the necessity for postmortem investigation in the SIDS diagnosis: “cases failing to meet the standards of this definition, including those without a post-mortem investigation, should not be diagnosed as SIDS. Cases that are autopsied and carefully investigated, but which remain unresolved may be designated as ‘undetermined,' ‘unexplained,' or the like” [2].

A further refinement of the definition of SIDS came with the 2004 “San Diego definition” [3]: “SIDS is defined as the sudden unexpected death of an infant <1 year of age, with onset of the fatal episode apparently occurring during sleep, that remains unexplained after a thorough investigation, including performance of a complete autopsy and review of the circumstances of death and the clinical history.” Thus, this definition adds that the death apparently occurred during sleep and that the evaluation of the circumstances of death is included. The San Diego definition is the currently used general definition of SIDS. Apart from this general definition, SIDS categories “IA,” “IB,” “II,” and “Unclassified” were defined for research purposes, where category IA is the most strict and limits the infant's age to more than 21 days and less than 9 months [3]. It is important to note that SIDS remains a diagnosis of exclusion in all definitions and that SIDS is not a syndrome with a single well-defined cause. A comprehensive review of the purposes for and challenges in defining SIDS has recently been provided by Krous [4]. 

Despite the above attempts in defining SIDS, the definitions and protocols used for diagnosing SIDS have never been fully standardized [4, 5]. This is illustrated by the audit of publications that was undertaken by Byard and Marshall [6]. They reviewed fifty papers dealing with SIDS that were published in 2005. In as many as 29 of these papers (58%), there was either no definition of SIDS or the definition was nonstandard or idiosyncratic. In the remaining 21 (42%) of the papers, one used the original Seattle definition, 15 (30%) used the NICHD definition, and 5 (10%) used the then recently published San Diego definition. As emphasized by Byard and Marshall [6], the evaluation of SIDS research may be severely hampered by the failure to use standard published definitions of SIDS and/or to clearly specify the definition that has been adopted. A further problem comes from the persistent inconsistency in the way pathologists report on sudden death in infants, as demonstrated by surveys among pediatric pathologists in the UK [7, 8].

In clinical practice, SIDS is specified as the cause of death through an International Classification of Diseases (ICDs) code on the death certificate, with its inherent shortcomings [9]. Currently, the Tenth Revision (ICD-10) is in use, with SIDS carrying the code R95, which was 798.0 in the Ninth Revision (ICD-9). A major update is to be realized with the introduction of the separate codes R95.0 (“sudden infant death syndrome with mention of autopsy”) and R95.9 (“sudden infant death syndrome without mention of autopsy”). This update has been approved by the World Health Organization in October 2009, with a suggested implementation date of January 2013.

SIDS is not to be confused with “sudden and unexpected death in infancy” (SUDI), or “sudden unexpected infant death” (SUID), which is a general term referring to all infant deaths that are sudden and unexpected, not just to those that meet the definition of SIDS [4, 10]. Even more than SIDS, SUDI is an umbrella label. As with SIDS, the use of the term SUDI is variable and efforts are undertaken to standardize the definition of SUDI [10–12]. A further term in use is postneonatal mortality (PNM), which simply refers to the death of liveborn infants from 28 through 364 days of age. It has been suggested that, given the variability and inconsistency in the assignment of the cause of infant death within and across countries, that the rate of PNM may be a better indicator of trends in both SIDS and other SUDI for comparative purposes [13].

### 1.2. Prevalence

**Figure 1:**
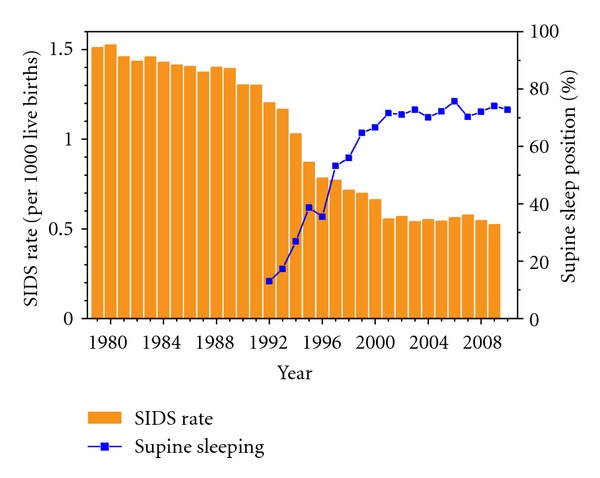
Rate of SIDS (orange bars), expressed as the number of SIDS cases per 1,000 live births, and the percent of infants put to sleep in the supine position (blue line with squares) in the United States over the period from 1979 to 2010. Data on SIDS rate (1979–2009) from the Centers for Disease Control and Prevention (CDC), as provided on the CDC Wide-ranging Online Data for Epidemiologic Research (WONDER) website (http://wonder.cdc.gov/mortSQL.html). For the years 1979–1999, the data refer to all deaths labeled with code 798.0 (“sudden infant death syndrome”) of the Ninth Revision of the International Classification of Diseases (ICD-9) of the World Health Organization. For the years 2000–2009, the data refer to all deaths specified with code R95 (“sudden infant death syndrome”) of the Tenth Revision (ICD-10). Data on supine sleep position (1992–2010) from the National Infant Sleep Position Study (NISP) of the National Institute of Child Health and Human Development (NICHD), as provided on the NISP public access website hosted by Boston University (http://slone-web2.bu.edu/ChimeNisp/Main_Nisp.asp).

The prevalence of SIDS varies considerably among countries, but a common trend is a significant decrease over time in the past decades. In the 1980s the rate of SIDS, expressed as the number of SIDS cases per 1,000 live births, varied around 1.4–1.5 in the United States (Figure 1, orange bars). In the 1990s, a remarkable decrease occurred, after which the SIDS rate stabilized at 0.5–0.6 per 1,000 live births in the 2000s. Nevertheless, SIDS has until today remained a major cause of infant mortality in the United States and other developed countries. Accounting for a total of 2,226 deaths in 2009, which implies that six infants died of SIDS per day, it was the third most important cause of infant mortality in the United States in 2009, only outranked by “congenital malformations, deformations and chromosomal abnormalities” (ICD-10 codes Q00–Q99) and “disorders related to short gestation and low birth weight, not elsewhere classified” (ICD-10 code P07) [14]. It is unlikely that the introduction of the ICD-10 code R95 in reporting SIDS in 2000 contributed to the observed decrease in SIDS rate. If anything, it was to be expected that SIDS would be selected as the underlying cause of death more often under ICD-10 than under ICD-9 [15].

 The sharp decrease in SIDS rate in the 1990s coincides with a sharp increase in the rate of supine sleeping, as determined from data collected in the National Infant Sleep Position Study (NISP) [16], which stabilized to *≈*72% in the 2000s (Figure 1, blue line with squares). This increase is considered a direct effect of the 1992 recommendation of the American Academy of Pediatrics to avoid babies to sleep in the prone position and the subsequent “Back to Sleep” campaign initiated in 1994, although a low adherence to sleep position recommendations among specific demographic groups, particularly among socioeconomically disadvantaged groups, persists [17–21]. The decrease in SIDS rate upon the increase in supine sleeping illustrates the significantly increased risk of SIDS of prone sleeping, as reviewed in several studies [21–24]. However, one should be careful to attribute all of the decrease in SIDS rates to the effects of the “Back to Sleep” campaign, since there is also a declining trend in diagnosing and reporting infant deaths as SIDS [25–27].

**Figure 2:**
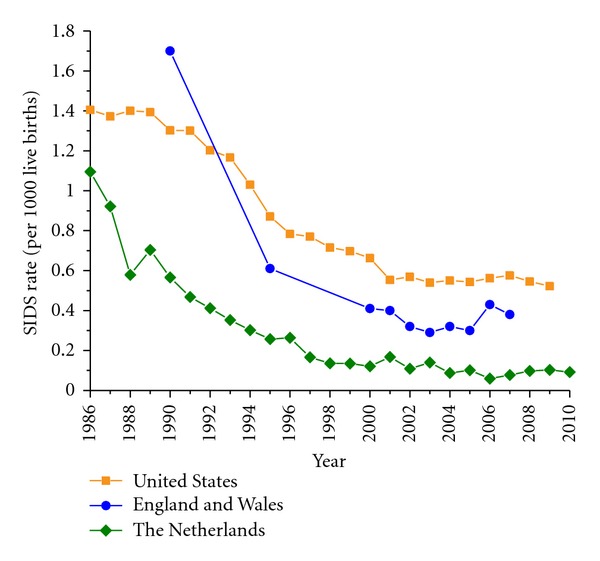
Rate of SIDS, expressed as the number of SIDS cases per 1,000 live births, over the period from 1986 to 2010 in the United States (orange solid line with squares), England and Wales (blue solid line with circles), and The Netherlands (green solid line with diamonds). Data on SIDS rate for the United States as in Figure 1. Data for England and Wales from Hauck and Tanabe [13] (1990–2005), completed with data for 2006 and 2007 from the International Society for the Study and Prevention of Perinatal and Infant Death (ISPID), as provided on the ISPID public access website (http://www.ispid.org/statistics.html). Data for The Netherlands from Statistics Netherlands (Centraal Bureau voor de Statistiek, CBS), as provided on the CBS public access website (http://www.cbs.nl/).

**Figure 3:**
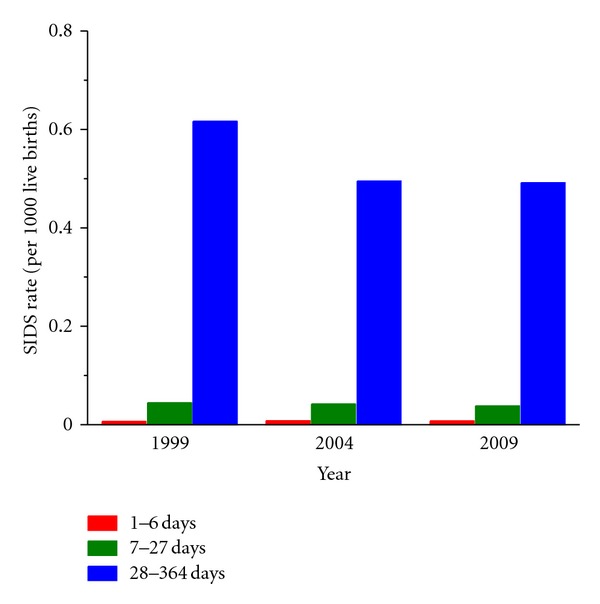
Rate of SIDS, expressed as the number of SIDS cases per 1,000 live births, occurring during week 1 of the infant's life (days 1–6, red bars), during weeks 2–4 (days 7–27, green bars), and during week 5 and later (days 28–364, blue bars) in the United States in 1999, 2004, and 2009. Data from the Compressed Mortality database as provided on the Wide-ranging Online Data for Epidemiologic Research (WONDER) website of the Centers for Disease Control and Prevention (CDC, http://wonder.cdc.gov/mortSQL.html).

 A similar or even higher decrease in SIDS rates in the 1990s is observed in other countries than the United States, where campaigns focusing on infants to be placed down for sleep in a nonprone position were initiated around 1990. In England and Wales, for example, where the national risk reduction campaign began in 1991, the SIDS rate dropped from 1.7 per 1,000 live births in 1990 to 0.41 per 1,000 live births in 2000 and stabilized to 0.3–0.4 in the 2000s [13] (Figure 2, blue line with circles). In The Netherlands, where the national risk reduction campaign began in 1987 [13], there was a decrease from 1.1 per 1,000 live births in 1986 to a steady 0.1 in the 2000s (Figure 2, green line with diamonds). This SIDS rate of 0.1 per 1,000 live births is among the lowest worldwide, together with that of Japan [13]. The differences in SIDS rates and PNM rates, between countries, as illustrated in Figure 2, is intriguing and has been discussed in detail elsewhere [13]. An obvious difference between countries is the age of inclusion for SIDS, which may be birth to one year, one week to one year, or three weeks to one year [13]. It is, however, unlikely that this creates large differences in SIDS rates across countries, because the far majority of SIDS cases occur after three weeks of age, as illustrated for the United States in Figure 3. In each of the years selected for Figure 3, >90% of the cases occurred after three weeks of age (Figure 3, blue bars). Although there have been some small shifts in the age of death over the years, the occurrence of SIDS remains rare during the first month of life (cf. Figure 3), increases to a peak between two and three months of age, and then decreases [28], as illustrated by the average age at the time of death of 2.9 ± 1.9 months in the Mayo Clinic cohort of 292 unrelated SIDS cases (see Section 4.2).

### 1.3. Common Risk Factors

In the previous section, prone sleeping was already addressed as an important, but fortunately avoidable, risk factor for SIDS. Related asphyxia generating risk factors are head covering [29, 30] and bed sharing [31, 32], which emerged as additional prominent risk factors after the “Back to Sleep” campaign had driven back the prone sleeping numbers [33]. Bed sharing is not to be confused with sharing the sleeping room at night with one or more adults, which rather considerably lowers the risk of SIDS [34].

 Another important risk factor for SIDS is smoking, both by the mother during pregnancy and in the household after birth [35, 36]. As with bed sharing, the attributed risk associating maternal smoking and SIDS has increased following the “Back to Sleep” campaign [37]. Interestingly, several studies have recently been undertaken in animal models to elucidate the mechanisms by which prenatal exposure to nicotine or postnatal exposure to cigarette smoke may contribute to SIDS [38, 39].

 Data on alcohol consumption as a risk factor for SIDS are, perhaps surprisingly, less clear, which may be due to the obscuring effect of its strong correlation with smoking, which makes it difficult to prove any additional independent effect [40]. In the study by Blair et al. [40], the SIDS victim mothers drank slightly more alcohol than controls, but these differences were not significant when adjusted for maternal smoking. McDonnell-Naughton et al. [41], however, recently reported that mothers of SIDS victims consumed significantly more alcohol during pregnancy than control mothers and that, within drinkers, the amount of alcohol consumed was also greater. Alm et al. [42], on the other hand, had found that heavy postnatal but not prenatal intake of alcohol by the mother increased the risk of SIDS.

As with alcohol consumption, data on maternal caffeine consumption throughout pregnancy as a risk factor for SIDS are not unequivocal. Again, there was a strong relation with smoking [42]. Whereas Ford et al. [43] found that, after adjustment for confounders, caffeine consumption of >400 mg/day (equivalent to four or more cups of coffee per day) throughout pregnancy significantly increased the risk of SIDS, such caffeine consumption during or after pregnancy was not found to be an independent risk factor for SIDS after adjustment for confounders by Alm et al. [42].

A further risk factor is found in child care settings [44, 45], with a significantly higher number of SIDS cases occurring in child care than would expected from the time spent there. There are no clear explanations why child care settings are a risk factor for SIDS. The current hypothesis is that changes in the routine infant care and resulting stress play an important role [44, 46]. These might cause sleep deprivation, leading to deeper sleep and impaired arousal [47].

Of note, each of the above risk factors for SIDS, that is, prone sleeping, head covering, bed sharing, smoking, and the consumption of alcohol and caffeine, is avoidable or modifiable. This is unfortunately not the case for all risk factors considered in the “triple risk model.”

### 1.4. Triple Risk Model

The presumably multifactorial nature of SIDS is reflected in the “triple risk model” that was introduced by Filiano and Kinney [48]. They proposed that SIDS can only occur if a vulnerable infant experiences one or more exogenous stressors during a critical developmental period in homeostatic control. Similar “triple risk hypotheses” had been put forward, as reviewed and discussed by Guntheroth and Spiers [49].

**Figure 4:**
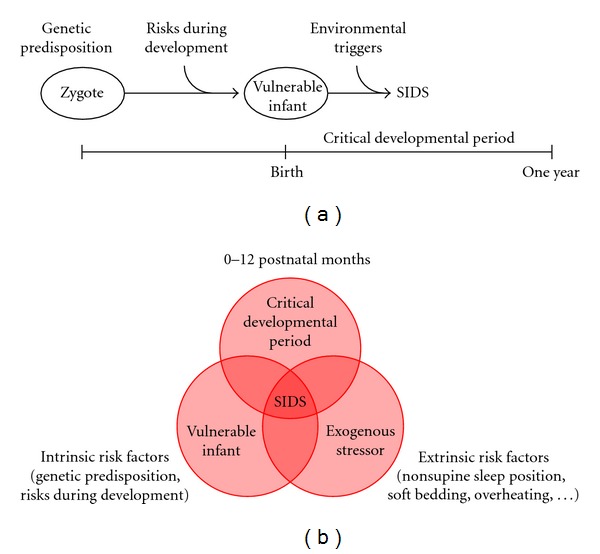
Triple risk model in the sudden infant death syndrome (SIDS). (a) Visualization of the triple risk model after Courts and Madea [50]. Genetic predisposition and risks during development create a vulnerable infant. If this vulnerable infant encounters environmental triggers during a critical developmental period, it may become an SIDS victim. (b) Visualization of the triple risk model after Filiano and Kinney [48] and Trachtenberg et al. [34]. A combination of intrinsic risk factors (genetic predisposition, risks during development) and extrinsic risk factors during a critical developmental period may create an SIDS victim.

Figure 4(a) illustrates the “triple risk model,” based on the visualization by Courts and Madea [50]. Genetic predisposition and risks during development, such as the aforementioned maternal cigarette smoking, create a vulnerable infant. If environmental triggers occur during a critical developmental period, SIDS may occur, typically between two and three months of age [28]. The “triple risk model” may also be visualized by means of a Venn diagram, as in Figure 4(b), which emphasizes, after Trachtenberg et al. [34], that SIDS tends to occur in the presence of a combination of “intrinsic risk factors” (genetic predisposition, risks during development) and “extrinsic risk factors” during a critical developmental period.

In line with the triple risk model, multiple intrinsic and/or extrinsic risk factors are found in the far majority of SIDS cases [34]. Here, an intrinsic risk factor is defined as “a genetic or environmental factor that affects susceptibility, including African American race, male gender, prematurity (<37 gestational weeks at birth), and prenatal maternal smoking or alcohol intake,” whereas an extrinsic risk factor is defined as “a physical stressor around the time of death that may increase the risk of SIDS for an already vulnerable infant,” such as an infection of the upper respiratory tract, which is relatively often observed in SIDS victims [34, 51].

 Of note, the triple risk model does not provide a single distinct explanation of SIDS. It rather underscores the multifactorial nature of SIDS, with genetic predispositions, known risk factors in development, and environmental triggers all playing their role. These environmental triggers include viral and bacterial infections [52–54].

### 1.5. Genetic Predispositions

Over the years, genetic predisposition to SIDS through mutations or (a combination of) polymorphisms has received wide attention. As reviewed by Blackwell et al. [55], several polymorphisms that facilitate uncontrolled inflammatory responses, in particular those resulting in the underproduction of the anti-inflammatory cytokine interleukin-10 (IL-10) or the overexpression of the proinflammatory cytokines IL-1*β* and IL-6, may be found at a higher proportion in SIDS victims than in controls. A genetic predisposition to elevated vascular endothelial growth factor (VEGF) levels may also play a role in SIDS. An association of SIDS with a VEGF gene polymorphism has been reported [56] and significantly higher VEGF concentrations were found in the cerebrospinal fluid of SIDS victims, pointing to a role for hypoxia in the cascade of events that lead to SIDS [57].

Also, polymorphisms in genes related to the autonomic nervous system have been associated with SIDS. Starting with the study by Narita et al. [58], several studies have demonstrated that SIDS victims are more likely than matched controls to have the long or extra-long allele of the *5HTT* serotonin transporter gene, which increases the effectiveness of the promoter and therefore of serotonin re-uptake by the 5-HT serotonin transporter, thus facilitating decreased serotonin concentrations at nerve endings and less effective protective responses to homeostatic challenges during sleep (but see Paterson et al. [59]). The “medullary serotonergic network deficiency hypothesis” and “brainstem hypothesis” have been reviewed elsewhere [60–62], with a particular emphasis on the role of abnormalities in the brainstem serotonin receptor binding, which is also a subject of one of the aforementioned animal studies on SIDS [38].

Further genetic factors related to the autonomic nervous system have been identified in SIDS research. Weese-Mayer et al. [63] analyzed several genes pertinent to early embryologic development of the autonomic nervous system. They identified rare protein-changing polymorphisms in association with SIDS in five genes (*PHOX2a*, *RET*, *ECE1*, *TLX3*, and *EN1*). After investigating left ventricular and blood samples from nine SIDS cases, Livolsi et al. [64] reported cholinergic abnormalities in the intracardiac part of the autonomic nervous system. Compared with controls, SIDS cases showed an increase in both the density of cardiac muscarinic receptors and the erythrocyte acetylcholinesterase enzyme activity. A subsequent study demonstrated that the cardiac muscarinic receptor overexpression plays a critical role in the development of vagal hyperreactivity, whereas the acetylcholinesterase hyperactivity appears as a compensatory consequence of it [65].

The above list of genetic factors associated with SIDS is far from complete and, for example, lacks any reference to polymorphisms related to glucose homeostasis that have been associated with SIDS [66–68]. However, it is my aim to provide a comprehensive overview of the genetic predisposition to SIDS that may result from mutations in cardiac ion channel-related genes. This overview will be given in subsequent sections, starting with the clinical associations that emerged in the 1970s.

## 2. Clinical Associations

In the 1970s and 1980s, possible associations of SIDS with cardiac disorders received a growing attention and were addressed in several studies, albeit with unequivocal results. It took until 1998 for conclusive data on the association of SIDS with a prolonged QT interval on the electrocardiogram (ECG), obtained in a 19-year prospective study, to become available. In the present section, the quest for clinical associations of SIDS with cardiac disorders is reviewed.

### 2.1. Cardiac Conduction Disorders and SIDS

Already in 1966, Fraser and Froggatt [69] suggested, in a “Letter to the Editor,” that “a proportion, probably small, of sudden unexplained deaths in infancy are due to genetically determined disorders of cardiac conduction.” Evidence for a role for cardiac conduction disorders in SIDS was later provided by Keeton et al. [70], who reported on six cases of “near-miss” sudden infant deaths in which severe conduction disorders where diagnosed and treated just before they became fatal. One may only speculate that in some of these cases the cardiac conduction disorder was genetically determined. In particular, mutations in the *SCN5A* gene, which encodes the pore-forming *α* subunit of the cardiac fast sodium channel, may result in the inhibition of the fast sodium current (*I*_Na_) and thus underlie conduction disorders. Such genetically determined cardiac conduction disorder (CCD), also designated “cardiac conduction defect” and “cardiac conduction disease,” has been reviewed in detail elsewhere [71–73].

### 2.2. Imbalance of Autonomic Tone and SIDS

In 1976, Schwartz [74] hypothesized that sympathetic imbalance may underlie SIDS. In particular, impaired right sympathetic activity would create such imbalance and thereby increase the likelihood of ventricular fibrillation. A clinical symptom might be QT interval prolongation on the ECG [74]. An imbalance of autonomic tone was also hypothesized by Montague et al. [75]. However, their data suggested an increase in sympathetic tone in infants at risk for SIDS. These data were obtained in a comparison of 17 infants at risk for SIDS and 17 age- and sex-matched control subjects. Their at-risk group consisted of 11 infants admitted to hospital for the investigation of unexplained apnea and six other infants, of whom five were subsequent siblings of SIDS patients and one was a sibling of a patient with near-miss SIDS. Significant cardiac dysrhythm and QT prolongation were not found in the at-risk infants. Rather, the rate-corrected QT interval (QTc interval) was consistently shorter in the at-risk group, indicative of increased sympathetic or decreased parasympathetic tone, of which an increase in sympathetic tone was considered the most likely [75].

 Further evidence for a role of a functional abnormality in the autonomic nervous system came from the study by Haddad et al. [76], who measured the QT interval in 7 near-miss SIDS victims and 12 control infants. Again, in contrast with the hypothesis of Schwartz [74], no QT prolongation was observed. The QT interval was even significantly smaller in the infants with aborted SIDS than in the control infants in both rapid eye movement (REM) and quiet sleep. These observations, together with data on heart rate and heart rate variability during sleep obtained at monthly intervals in 18 control infants and 12 infants with aborted SIDS during the first four months of life, led Leistner et al. [77] to conclude that the infants with aborted SIDS had an increase in sympathetic activity or in circulating levels of catecholamines.

### 2.3. Long QT Syndrome and SIDS

The long QT syndrome (LQTS) is characterized by QT prolongation on the ECG and a predisposition to syncope, seizures, and sudden cardiac death, caused by episodic polymorphic ventricular tachyarrhythmias such as Torsade de Pointes. These and other (clinical) features of LQTS have been reviewed in detail elsewhere [78, 79]. Especially in young people, LQTS is an important cause of sudden death. Of note, all features of LQTS, including a negative postmortem examination, are compatible with SIDS, which makes LQTS a likely cause of SIDS.

#### 2.3.1. Unequivocal Observations in the 1970s and 1980s

**Figure 5:**
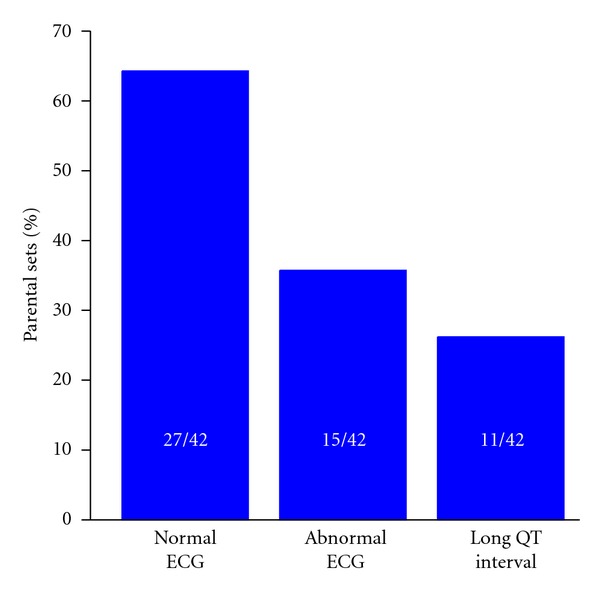
Electrocardiographic data in 42 sets of parents who had infants with SIDS [80]. In 27 sets of parents, both parents had a normal ECG (left bar). In 15 sets (36%, middle bar), at least one parent had an abnormal ECG (middle bar), including a long QT interval in 11 sets (26%, right bar).

In 1976, Maron et al. [80] proposed inheritable LQTS as a possible cause of SIDS. They obtained electrocardiographic data from 42 sets of parents who had at least one infant with SIDS and found that a considerable proportion of first-degree relatives of infants with SIDS had a prolongation of the QT interval on the ECG. In 27 sets of parents, the ECG was normal for both parents, as illustrated in Figure 5. However, at least one of the parents showed an abnormal ECG in the remaining 15 sets. This included QT prolongation in 11 of these sets, which is in 26% of the 42 sets studied (Figure 5). To investigate the possible genetic transmission of the prolonged QT interval, ECGs were taken from 23 siblings of infants with SIDS. All of these siblings were members of eight families in which at least one of the parents had a prolonged QT interval. The ECGs showed the prolongation of the QT interval in 9 of the 23 siblings (39%), whereas none of the 18 siblings of infants with SIDS from seven families in which neither parent had a prolonged QT interval showed QT interval prolongation. As a further evidence for an association between SIDS and a prolonged QT interval, Maron et al. [80] also described the case of an infant with near-miss SIDS, who survived a cardiorespiratory arrest at seven weeks of age and showed marked prolongation of the QT interval. However, her parents did not show prolongation of the QT interval, nor did 16 other family members except a 10-month-old nephew with a mildly prolonged QT interval. Tragically, having been in apparent excellent health for twelve years, she still died unexpectedly in her sleep, suggesting “the possibility that “near-miss” episodes in infancy may infer a long-term risk that has not been appreciated” [81]. Of note, Maron et al. [80] emphasized that their results were not definitive and that the confirmation of a relation between QT interval prolongation and SIDS would require large prospective investigations.

Although the results of Maron et al. [80] strongly suggested a role for QT interval prolongation in SIDS, one should realize that no such data were obtained in several other studies, including some discussed in the previous section. In 1973, Froggatt and James [82] already postulated a role for “aberrant ventricular repolarization expressed through a prolonged QT interval” in SIDS, but such a role could not be established from their data collected from a total of 162 SIDS cases over the years. The families were visited in 148 cases and the ECGs obtained from 238 of possible 296 parents. These ECGs revealed no significant arrhythmias and the number with a prolonged QT interval was not enhanced as compared to what would be expected from natural variation. Also, none of the parents had a history of pertinent syncopal attacks. In 1977, Kelly et al. [83] reported the results of their review of the postresuscitation ECGs obtained from 21 near-miss SIDS infants to evaluate the role of QT interval prolongation in the genesis of SIDS. The QT intervals were not significantly different from those of age- and sex-matched controls. Also in 1977, Kukolich et al. [84] reported that ECG studies in a total of 108 first-degree relatives of 26 SIDS victims in comparison with 99 such subjects from 22 control families failed to show any significant differences in the QT interval between these two groups. In 1978, Steinschneider [85] reported data on the QTc interval of 30 neonates whose sibling had died of SIDS in comparison to that of 75 control neonates. The QTc interval of siblings of SIDS victims did not differ from that of the control infants. Also, data were available on the QTc interval of 52 parents of an SIDS victim. No prolongation of the QT interval was apparent in this group. In a subsequent study, Weinstein and Steinschneider [86] found that none of the eight infants who subsequently died of SIDS had a prolonged QTc interval. However, the RR interval during REM sleep was significantly shorter in these future SIDS victims than in control subjects, as in the aforementioned study by Leistner et al. [77] on infants with aborted SIDS. Finally, Southall et al. [87] recorded standard ECGs from a total of 7,254 newly born infants from two maternity hospitals. Fifteen of these infants subsequently suffered SIDS (corresponding to an SIDS rate of 2.1 per 1,000 live births in this cohort). However, no significant differences could be identified in their QTc intervals compared to controls in age matched-groups or after individual matching for postnatal age, hospital of birth, and weight at birth. This was in line with the results of a previous study by Southall et al. [88] on 1,157 preterm and/or low-birth-weight infants within one week prior to discharge from eight neonatal intensive care units. Five of these infants became SIDS victims (corresponding to an SIDS rate of 4.0 per 1,000 live births in this at-risk cohort). Southall et al. [88] made 24-h recordings of beating movement and ECG and found that a proportion of apparently well infants had prolonged apnea, extreme bradycardia, or cardiac arrhythmias. However, none of these disorders was predictive of the five subsequent cases of SIDS. A similar, one-year-later study [89] included 6,914 full-term and 2,337 preterm and/or low-birth-weight infants, of whom 29 (17 full term, 12 preterm) subsequently suffered SIDS (corresponding to SIDS rates of 2.5 and 5.1 per 1,000 live births in the full-term and preterm cohorts, resp.). Again, no abnormal prolongation of the QT interval was observed.

Thus, the initial study by Maron et al. [80] was followed by a series of negative data on an association between LQTS and SIDS [82–89]. At the same time, some supporting evidence was also obtained. In 1979, Southall et al. [90] reported a case of a neonate who had suffered from arrhythmias in utero and bradycardia for the first 9 days of life. A normal heart rate was documented at 10 days and he was discharged from hospital, but died suddenly and unexpectedly three days later, the negative postmortem examination classifying him as an SIDS victim. A retrospective analysis of the perinatal ECG revealed a substantially prolonged QTc interval. A second neonate had also suffered from arrhythmias in utero. She showed a prolonged QTc interval and frequent premature ventricular beats on a 24-hour ECG, but she was successfully treated with the *β*-blocker propranolol. In 1982, a prospective study by Schwartz et al. [91] was published. This study was specifically designed to test the “QT hypothesis” for SIDS. Standard ECGs were recorded from 4,205 newborns on the fourth day of life and for some at later stages as well. Of the 2,000 infants checked at one year, three had become SIDS victims (corresponding to an SIDS rate of 1.5 per 1,000 live births in this cohort). One had a clearly prolonged QTc interval of 563 ms on the ECG recorded on the fourth day of life, whereas the other two also showed signs of a prolonged QTc interval. Sadeh et al. [92] studied the dependence of the QT interval on the preceding RR interval in 10 infants with SIDS and 29 healthy control infants, analyzing *≈*5,000 pairs of QT and RR intervals in each subject over a wide range of RR intervals. They found an impaired dependence, that is, an impaired ability to shorten the QT interval as the heart rate increased, in 5 of 10 infants who subsequently died of SIDS. There are, however, some serious methodological shortcomings in the study by Sadeh et al. [92], as set out by Guntheroth [93] in a critical review of the possibility that SIDS is due to a primary cardiac disorder, in which he concluded—at the end of the 1980s—that “there is no statistical basis, with sound and reproducible methods, to support a cardiac theory for the cause of sudden infant death syndrome.”

#### 2.3.2. Conclusive Data in the 1990s

As set out in the previous section, numerous studies carried out in the 1970s and 1980s addressed the issue of a relationship between LQTS and SIDS. Unfortunately, the data that were available by the end of the 1980s were far from conclusive, as illustrated by the aforementioned critical review by Guntheroth [93]. This changed with the publication in 1998 of a hallmark paper by Schwartz et al. [94], in which they reported results from a 19-year electrocardiographic assessment of Italian neonates at day three or four of life. Of the 34,442 infants enrolled, one-year follow-up data were available for 33,034. In this cohort, 24 infants died of SIDS (corresponding to an SIDS rate of 0.73 per 1,000 live births in this cohort). The mean QTc interval of the SIDS victims was significantly longer than that of infants who survived or died of other causes. More importantly, in 12 of them the QTc interval was considered to be prolonged (>440 ms), whereas it was not in any of the 10 infants who died of other causes. It was found that the odds ratio for SIDS in infants with a prolonged QTc interval in the first week of life was as high as 41.3. It remains to be elucidated why no indications for such strong association had been obtained in earlier prospective studies [87–89].

In Italy, as a followup of the study by Schwartz et al. [94], a large prospective ECG study in a population of as many as 44,596 neonates, in whom an ECG was recorded between the 15th and the 25th day of life to identify infants with LQTS and thus at risk for SIDS, was recently completed [95]. In 28 of the 29 infants with marked QT interval prolongation (QTc interval >470 ms), a molecular screening for mutations in known “LQTS genes” (see Section 3.1 below), was performed. This screening revealed LQTS mutations in 12 neonates. Another four mutation carriers were identified upon genetic analysis of 14 of the 28 neonates with a QTc interval between 461 and 470 ms. All but one, because of parental refusal, of the 29 neonates with a QTc interval >470 ms were successfully treated with a *β*-blocker (propranolol). It is tempting to speculate that this treatment prevented the occurrence of SIDS in this group.

## 3. Primary Electrical Cardiac Diseases

As we know now, LQTS is an inheritable disorder, caused by mutations in genes predominantly encoding subunits of cardiac ion channels [96, 97]. If, for example, such mutation causes a “loss of function” of potassium channels that carry an outward current that contributes to the repolarization of the cardiac action potential, a delay in repolarization and prolongation of the QT interval results. LQTS is considered a “primary electrical cardiac disease,” with arrhythmogenic events generated at the cellular or multicellular level due to disturbed ion channels. As such, LQTS and other “ion channelopathies” may well underlie cases of SIDS, because they are not detectable during a standard postmortem examination. This does not only hold for LQTS, but also for other primary electrical cardiac diseases, in particular the short QT syndrome (SQTS), the Brugada syndrome (BrS), and cathecholaminergic polymorphic ventricular tachycardia (CPVT), which share several clinical features with LQTS, such as a predisposition to sudden cardiac death. With their primary electrical nature, they should be taken into account in relation to SIDS.

### 3.1. Long QT Syndrome

**Figure 6:**
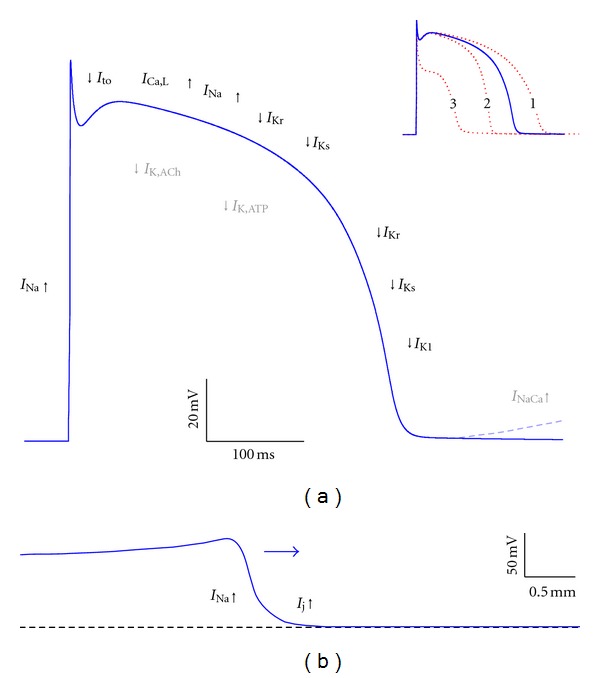
Action potential formation and propagation in ventricular myocytes. (a) Diagram of an action potential in an isolated ventricular myocyte with individual ion currents that “push up” (inward currents, upward arrows) or “pull down” (outward currents, downward arrows) the action potential. *I*_Ca,L_: L-type calcium current; *I*_K,ACh_: acetylcholine-sensitive potassium current; *I*_K,ATP_: ATP-sensitive potassium current; *I*_K1_: inward rectifier potassium current; *I*_Kr_: rapid delayed rectifier potassium current; *I*_Ks_: slow delayed rectifier potassium current; *I*_Na_: fast sodium current; *I*_NaCa_: sodium-calcium exchange current; *I*_to_: transient outward potassium current. Grey symbols refer to currents that become important under special conditions (release of ACh, shortage of ATP and calcium overload for *I*_K,ACh_, *I*_K,ATP_, and *I*_NaCa_, resp.). Loss of function of “pull down” currents may result in action potential lengthening (inset, red dashed trace labeled “1”), whereas loss of function of “push up” currents may result in action potential shortening (inset, red dashed trace labeled “2”) and loss of the action potential dome (inset, red dashed trace labeled “3”). (b) Diagram of action potential propagation. The propagating action potential (horizontal arrow) brings neighboring cells to threshold through the *I*_Na_ driven gap junctional current *I*_j_.

At the cellular level, LQTS is characterized by an increase in action potential duration, which may result from an increase in inward current during the plateau phase of the action potential, for example, due to an increase in the late component of the fast sodium current (*I*_Na_; “gain of function”), or a decrease in outward current, for example, due to a decrease in the rapid or slow delayed rectifier potassium current (*I*_Kr_ and *I*_Ks_, resp.; “loss of function”), as illustrated in Figure 6(a).  Table 1 provides an overview of the mutations in ion channel-related genes that have been associated with LQTS. For example, mutations in *KCNH2 *and* KCNE2*, encoding the *α* and *β* subunits of the *I*_Kr_ channel and classified as LQTS types 2 (LQT2) and 6 (LQT6), respectively, may both result in loss of function of the *I*_Kr_ channel, thereby prolonging the action potential and the QT interval. LQTS-related mutations have not only been found in *α* and *β* subunits of ion channels, but also in genes encoding anchoring and scaffolding proteins that affect the function of ion channels. This is the case in the (rare) LQTS types 4, 9, 11, and 12 (Table 1).

 Inheritance of LQTS is autosomal dominant except for the rare autosomal recessive variant that is known as the Jervell and Lange-Nielsen syndrome [98, 99]. In this syndrome, autosomal recessive mutations in *KCNQ1* or* KCNE1*, encoding the *α* and *β* subunits of the *I*_Ks_ channel, respectively, cause severe QT prolongation [100, 101]. The syndrome is also associated with inner ear deafness, which is explained by the critical role of *I*_Ks_ channels in the secretion of potassium ions into the endolymph [102]. Of note, other rare types of LQTS, in particular LQTS types 7 and 8 (LQT7 and LQT8), are also associated with extracardiac symptoms. In LQT7, which is also known as the Andersen-Tawil syndrome [103, 104], mutations in the *KCNJ2 *gene result in disturbed K_ir_2.1 channels, which are expressed in multiple organs. LQT7 is not only characterized by (mild) QT prolongation and other ECG abnormalities, but also by periodic paralysis and dysmorphologies [105–107]. In LQT8, which is also known as the Timothy syndrome [108, 109], mutations in the *CACNA1C *gene result in disturbed C_av_1.2 channels [110]. Expression of these calcium channels is not limited to the heart. As a result, LQT8 is not only characterized by QT prolongation and other cardiac symptoms, but also by syndactyly of fingers and toes, intermittent hypoglycemia, immune deficiency, cognitive abnormalities, and autism [108, 111].

 The aforementioned recently completed study by Schwartz et al. [95] revealed 29 infants with marked QT interval prolongation (QTc interval >470 ms). In 28 of these 29 infants, a molecular screening for mutations in any of the LQTS genes *KCNQ1*, *KCNH2*, *SCN5A*, *KCNE1*, *KCNE2*, *CAV3*, and *SCN4B*, associated with the LQTS types 1–3, 5, 6, 9, and 10, respectively (Table 1), was performed. LQTS mutations were identified in 12 neonates. Another four mutation carriers were identified upon genetic analysis of 14 of the 28 neonates with a QTc interval between 461 and 470 ms. From the number of 17 of 43,080 white infants affected by LQTS—16 with disease-causing mutations and one with a clear-cut clinical diagnosis—one arrives at an LQTS prevalence of *≈*1 : 2,500 apparently healthy live births [95]. Given that infants with a QTc interval >450 ms were not molecularly screened and that the same holds for silent mutation carriers, that is, infants who carry a disease-causing mutation but showed a normal QT interval, the actual prevalence of LQTS may be around 1 : 2,000 [97]. However, the prevalence of LQTS remains a matter of debate, with on the one hand data suggesting an underestimation [112] and on the other hand data suggesting an overestimation [113].

In the far majority of clinically definite LQTS cases mutations in LQTS genes are found, with *KCNQ1*  (LQT1),*KCNH2*  (LQT2), and*SCN5A*   (LQT3) as the most common LQTS genes, accounting for *≈*90% of all genotype-positive cases [97, 114]. Gain-of-function mutations in the cardiac sodium channel gene *SCN5A* cause LQTS, due to a persistent inward sodium current during the plateau phase of the action potential, which is also known as “late” or “sustained” sodium current (Figure 6(a)). Although defects in *SCN5A* account for only *≈*10% of LQTS cases [115, 116], this type (LQT3) plays a rather important role in the etiology of LQTS. It appears that patients with LQT3 have significantly more severe clinical events than patients with LQT1 or LQT2, as the overall number of cardiac deaths is similar for these subgroups while the frequency of events is lower in LQT3 [117]. More specifically, severe symptoms are common in children with LQT3 [118]. This discrepancy between prevalence and severity should be taken into account when considering mutations in LQTS genes in relation to SIDS.

### 3.2. Brugada Syndrome

Mutations in cardiac ion channel genes may also lead to the Brugada syndrome (BrS) [119–122], which shares several clinical features with LQTS, such as a predisposition to sudden cardiac death. In BrS patients sudden death often occurs during rest or while sleeping, which makes BrS a likely cause for SIDS. BrS is characterized by changes in the ST segment of the ECG rather than QT prolongation, which may result from transmural dispersion in action potential duration, in particular in the right ventricle, due to an early repolarization in the epicardial cell layers. This has been explained on a cellular basis by the loss of the action potential dome in cells with a large transient outward potassium current, like the right ventricular epicardial cells, due to a decrease in the peak component of *I*_Na_ as a result of a loss-of-function mutation in *SCN5A* [123]. It should, however, be noted that this explanation of BrS as a repolarization disease is a matter of debate and that it may also, or at the same time, be a depolarization disease [124, 125]. The exact pathophysiological mechanism remains elusive and may involve structural abnormalities that are not easily detectable [126], but as far as ion channel disturbances are involved, these have as a common functional effect that they decrease the net inward current during the rapid depolarization phase of the action potential and/or increase the net outward current during the subsequent early repolarization phase (Table 2, Figure 6(a)). Several genes have been linked to BrS, but in some the associated mutations are not universally considered pathogenic, as reflected in literature where different genes can be found in relation to BrS types 8 and higher.

The prevalence of BrS is not well established, but may be somewhere between 1 : 2,000 and 1 : 5,000, with a higher prevalence in South-East Asia [127]. In contrast with LQTS, where ion channel-related mutations are identified in the far majority of clinically definite cases, BrS can be less well genotyped. Crotti et al. [128] recently assessed the presence of putative pathogenic mutations in each of the BrS-related genes *SCN5A*, *GPD1-L*, *CACNA1C*, *CACNB2b*, *SCN1B*, *KCNE3*, *SCN3B*, *HCN4*, *CACNA2D1*, *MOG1*, *KCND3*, *KCNJ8*, and *SCN1Bb* in a cohort of 129 unrelated patients referred for BrS genetic testing. These are all of the 14 genes listed in Table 2 except *KCNE1L*. Variants of the latter gene, which is also known as *KCNE5*, have been reported as BrS modulators by Ohno et al. [129]. Crotti et al. [128] identified mutations in 27 patients (21%). Most (21, or 16%) had a mutation in BrS1 gene *SCN5A*, in line with the study by Kapplinger et al. [130], who found an overall yield of 21% BrS1-associated *SCN5A* mutations derived from over 2,100 unrelated patients referred for BrS genetic testing, with the yield of the nine centers involved ranging between 11% and 28%. Crotti et al. [128] identified six patients (4.6%) with a mutation in one of the 12 other genes tested: two with a mutation in* CACNB2b* and one with a mutation in each of* HCN4*, *KCND3*, *KCNJ8,* and *SCN1Bb*. The low <2% yield of mutations involving the L-type calcium channel genes *CACNA1C*, *CACNB2b,* and *CACNA2D1* is in contrast with previous findings of 8.5% for *CACNA1C* and *CACNB2b* only [131] and 12.3% for *CACNA1C*, *CACNB2b,* and *CACNA2D1* all three [132] and may be related to the presence of a concomitant short QT interval [128], as in the study by Burashnikov et al. [132] and also reported by Hong et al. [133].

Overall, ion channel-related mutations are found in a minority of BrS patients, and if such mutation is found, it is almost always a mutation in *SCN5A* (BrS1). Mutations in other genes are rare and have thus far only been identified in single patients or in small families, so that one should be cautious with inferring these genes in the pathogenesis of BrS, as illustrated by the recent finding by Holst et al. [134] that support the association of *SCN1Bb* with BrS, but challenge that of *SCN3B*, *MOG1,* and *KCND3*. Many of the BrS-associated mutations listed in Table 2 have only recently been identified. This holds in particular for the mutations in *CACNA2D1* [132], *MOG1* [135], *KCND3* [136], *KCNE1L* (*KCNE5*) [129], *KCNJ8* [137], and *SCN1Bb* [138] and explains why there is no consensus in allocating BrS types 8 and higher to specific genes.

### 3.3. Short QT Syndrome

In contrast with LQTS, the short QT syndrome (SQTS) is characterized by a shortening of the QT interval on the ECG and, at the cellular level, a shortening of the action potential [139–141]. This shortening may be due to gain-of-function mutations in genes related to outward currents that flow during the repolarization phase of the action potential, for example, *I*_Kr_, *I*_Ks_, and *I*_K1_ (SQTS types 1–3; Table 3), or loss-of-function mutations in genes related to inward currents flowing during the repolarization phase, in particular the L-type calcium current, *I*_Ca,L_ (SQTS types 4–6; Table 3). SQTS is a relatively recent addition to the family of inheritable arrhythmogenic diseases. After reports on familial SQTS by Gussak et al. in 2000 [142] and by Gaita et al. in 2003 [143], disease-causing mutations in *KCNH2* (SQTS type 1, STQ1) [144], *KCNQ1* (STQ2) [145], *KCNJ2* (STQ3) [146], and *CACNA1C *and*CACNB2b* (STQ4 and STQ5) [131] were reported in 2004–2007. Recently, a sixth SQTS entity, associated with mutations in *CACNA2D1*, was reported by Templin et al. [147].

Although data on the prevalence of SQTS are limited [148–150], it is clear that the prevalence of SQTS is much lower than that of LQTS and BrS. However, SQTS carries a high risk of sudden death and may be a cause of death in early infancy [139], thus possibly underlying cases of SIDS.

### 3.4. Cathecholaminergic Polymorphic Ventricular Tachycardia

Catecholaminergic polymorphic ventricular tachycardia (CPVT) is a relatively rare but highly malignant inheritable arrhythmogenic disease. It is characterized by adrenergically mediated polymorphic ventricular tachyarrhythmias in the absence of electrocardiographic markers and structural heart disease and is induced by catecholamines released during physical exercise or emotional stress [151–155]. Thus far, mutations in three genes have been linked to CPVT, autosomal dominant mutations in the *RYR2 *gene encoding for the cardiac ryanodine receptor isoform 2 (CPVT type 1 or CPVT1) and rare autosomal recessive mutations in the *CASQ2 *and *TRDN *genes, encoding for the cardiac calsequestrin isoform 2 (CPVT2) and the triadin protein, respectively. All three proteins are involved in the intracellular cardiac calcium homeostasis (Table 4). The associations with the *RYR2 *and *CASQ2 *genes were both reported in 2001 [156, 157], whereas that with the *TRDN *gene was only recently reported [158]. In 2007, another autosomal recessive form of CPVT had been reported by Bhuiyan et al. [159], which mapped to a 25 Mb interval on chromosome 7p14–p22, but at present the causal gene at this locus is unknown. The *TRDN *variant is not listed as “CPVT3” in Table 4, because CPVT3 has already been used in relation to the study by Bhuiyan et al. [159] (OMIM entry %614021).

The calcium leak from the sarcoplasmic reticulum associated with the *RYR2 *mutations increases the probability of aberrant RyR2 channel opening during diastole [160]. This may result in delayed afterdepolarizations or spontaneous depolarization during diastole, which are both triggers for arrhythmias, due to the activation of the sodium-calcium exchange current *I*_NaCa_, as illustrated in Figure 6(a). As a paradigm to understand the mechanisms of arrhythmias associated to impaired Ca^2+^ regulation [153], CPVT has been widely studied in mouse models [160–163], identifying Purkinje cells as critical contributors to arrhythmic triggers in CPVT and suggesting a broader role for the Purkinje fiber network in the genesis of ventricular arrhythmias [163].

The prevalence of CPVT in the population is not known, but it has been estimated around 1 : 10,000 [151], which is probably an underestimation, as set out by de la Fuente et al. [164]. The potentially lethal arrhythmias associated with CPVT mostly occur in children and teenagers and are typically triggered by stress or exercise. Because sudden cardiac death may be the first manifestation of CPVT, it may well underlie cases of SIDS.

### 3.5. Caveat

In the above, the set of “primary electrical cardiac diseases” has been limited to LQTS, BrS, SQTS, and CPVT, as in many other papers [165–170] in which inheritable arrhythmogenic diseases are categorized and/or opposed to inheritable cardiomyopathies due to mutations in genes encoding sarcomeric or cytoskeletal proteins. There are, however, more electrical diseases, like the aforementioned inherited cardiac conduction disease [71] and the early repolarization and J wave syndromes [132, 137]. The electrical diseases are not separate entities, but may show overlap, not only in clinical symptoms but also in the underlying mutations [72, 171]. The latter is perhaps best illustrated by the 1795insD mutation in *SCN5A*, which has been associated with sinus node dysfunction, bradycardia, conduction disease, BrS, and LQTS, either in isolation or in combinations thereof [172–174]. Similar overlap syndromes have been reported for other mutations, in particular mutations in *SCN5A*, like E161K [175]. In addition, incomplete penetrance and variable expressivity may be observed [176], with family members with the same mutation demonstrating widely different clinical symptoms.

Furthermore, arrhythmogenic diseases may have some characteristics of cardiomyopathies and vice versa. As mentioned before, the pathophysiological mechanism of BrS may involve structural abnormalities that are not easily detectable [126], which would make BrS not purely an electrical disease. On the other hand, mutations in “structural genes,” as they occur in inheritable cardiomyopathies, may lead to a reduction in sodium current and intercellular coupling, thereby creating an arrhythmogenic substrate that is largely of an electrical nature. This may in particular hold for the early phase of arrhythmogenic right ventricular cardiomyopathy (ARVC; see [177] and primary references cited therein), which was initially termed arrhythmogenic right ventricular dysplasia (ARVD) or arrhythmogenic right ventricular dysplasia/cardiomyopathy (ARVD/C) and is currently being termed “arrhythmogenic cardiomyopathy” or “arrhythmogenic ventricular cardiomyopathy,” taking into account that the disease may also affect the left ventricle.

 Overall, ion channel-related mutations associated with primary electrical cardiac diseases, as listed in Tables 1–4, should be taken into account in relation to SIDS. In addition, there may be early phases of genetically determined cardiomyopathies that cannot be identified macroscopically by autopsy, but add to the vulnerability of the infant and contribute to SIDS, as recently reported for hypertrophic cardiomyopathy (HCM) and HCM-associated genes [168, 178]. Disruption of sarcomeric activity might in turn disrupt intracellular calcium homeostasis and thus be responsible for arrhythmogenesis [178].

## 4. Cardiac Ion Channelopathies in Molecular Autopsy

In 2000 and 2001, a number of case reports were published on mutations in LQTS genes in SIDS or near SIDS [179–181], starting with the description by Schwartz et al. [179] of an infant who nearly died of SIDS and in whom LQTS was diagnosed and a spontaneous mutation in *SCN5A* was identified, providing a “proof of concept” of cardiac ion channelopathies as a cause of SIDS. Shortly thereafter, Ackerman et al. [182] reported the results of the first population-based “molecular autopsy” study, screening for mutations in LQTS genes as the possible cause of SIDS. A series of such population-based cohort studies of SIDS cases followed. These postmortem genetic analyses, screening for mutations in LQTS, SQTS, BrS, and CPVT genes, have revealed associations of mutations in these genes with SIDS. In the present section, I will first review the proof-of-concept findings and next the outcome of the various genetic analyses of population-based SIDS cohorts.

### 4.1. Proof-of-Concept Findings

In 2000, a “proof of concept” of cardiac ion channel mutations as a possible cause of SIDS was provided with the aforementioned description by Schwartz et al. [179] of an infant who was resuscitated from ventricular fibrillation and in whom LQTS was diagnosed and a de novo missense mutation in *SCN5A* (S941N) was identified. In another two case reports, mutations were identified in the *KCNQ1* and *SCN5A* genes [180, 181]. The P117L mutation in *KCNQ1* was identified in an SIDS victim upon genetic analysis and appeared a de novo missense mutation that had already been identified in an unrelated family that was affected by LQTS [180]. The A1330P mutation in *SCN5A* also appeared a de novo missense mutation, resulting in a functionally disturbed hyperactive *I*_Na_ channel, as demonstrated in voltage clamp recordings from HEK-293 cells expressing the mutant channels, which showed a positive shift in voltage dependence of inactivation, a slowing of the time course of inactivation, and faster recovery from inactivation [181]. Effects of the mutation on the ventricular action potential, including a significant prolongation, were later directly demonstrated in “dynamic action potential clamp” experiments [183]. It is likely that the mutation caused the prolonged QTc interval of 600 ms on the ECG taken on day 5 as well as the lethal tachyarrhythmia in week 9 that occurred despite propranolol therapy. Sadly, cardiopulmonary resuscitation was not successful.

Several other case reports on ion channel mutations in SIDS or near SIDS have appeared in the literature since the reports by Schwartz et al. [179, 180] and Wedekind et al. [181]. A Scandinavian group found a novel mutation in *KCNH2* (K101E) in a seven-week-old SIDS victim [184]. Because of a positive family history for clinical LQTS and documented Torsade de Pointes, it was assumed that this mutation may well have caused a lethal arrhythmia. Nof et al. [185] presented a family in which an inherited common polymorphism in *KCNH2* (K897T) combined with a loss-of-function mutation (P926AfsX14) on separate alleles of the same gene led to sudden infant death and spontaneous abortion. Family members with only the polymorphism or only the mutation did not have any events of syncope or sudden cardiac death. Coexpression studies demonstrated a much greater loss of the function of *KCNH2 *current in the case of P926AfsX14/K897T than for P926AfsX14 or K897T alone.

Further case reports have all dealt with mutations in *SCN5A*. Skinner et al. [186] reported a near-miss SIDS case of a 19-day-old infant with documented marginal QTc interval prolongation and ventricular fibrillation. Genetic analysis revealed a missense mutation in *SCN5A* (R1193Q) that had been associated with BrS in a previous study [187] and had been characterized as an LQT3 mutation by Wang et al. [188]. Turillazzi et al. [189] linked the heterozygous W822X nonsense mutation in *SCN5A* to the death of both members of a set of apparently healthy monozygotic twins (“simultaneous sudden infant death syndrome,” SSIDS). This mutation had been linked to BrS in a previous study [190]. Huang et al. [191] described the case of a de novo mutation in *SCN5A* (S1333Y) identified in an SIDS victim who also carried a T20I mutation in *KCNE1*. Priori et al. [192] reported on two cases of SIDS in a family with BrS and the L567Q mutation in *SCN5A*, which was later functionally characterized as a loss-of-function mutation by Wan et al. [193]. Although direct genetic data of the two victims are not available, it is likely that they carried the familial L567Q mutation. Chockalingam et al. [194] described how an apparently healthy 4-month-old girl almost became an SIDS victim when immunization and/or the subsequent fever triggered life-threatening arrhythmias. Molecular analysis revealed a novel splice-site mutation in* SCN5A* that was then also identified in her father and in her 3-year-old brother. On the basis of the combination of findings in different family members, a diagnosis of BrS could be made, associated in this case with significant conduction defects.

### 4.2. Molecular Autopsy of Population-Based SIDS Cohorts

Starting with the study by Ackerman et al. [182] a series of population-based cohort studies have been published in which postmortem genetic analysis of SIDS cases, screening for mutations in LQTS genes and related genes (“molecular autopsy”), has revealed associations of mutations or rare variants in these genes with SIDS. The results of these studies are briefly discussed in order of publication below and summarized in Table 5.

Ackerman et al. [182] genetically analyzed postmortem cardiac tissue from 45 SIDS and 48 possible SIDS cases, obtained between September 1997 and August 1999 in the State of Arkansas, USA, for mutations in *SCN5A*. In two of these 93 cases, a missense mutation in *SCN5A* was found (A997S and R1826H). In either case, the mutant channels were investigated in an expression system, where the mutant sodium current showed a slower decay and a two- to threefold increase in late current, compatible with LQTS type 3 (LQT3). The two mutations are therefore listed in bold in Table 5, indicating that they are functionally of a malignant nature. The same cohort was used by Tester and Ackerman [195] when screening for mutations in *KCNQ1*, *KCNH2*, *KCNE1,* and *KCNE2*. They found novel potassium channel variants in* KCNQ1* (T600M), *KCNH2* (G294V), and *KCNE2 *(V14I), as listed in Table 5. In addition, they discovered a novel mutation in* KCNH2* (P1157L) following postmortem genetic testing in a separate case of an infant who was found dead in the prone sleep position. None of these mutations was seen in nearly 1,500 reference alleles from healthy controls. However, there is no functional evidence, for example, from in vitro expression studies, that any of these four mutations leads to potassium channel dysfunction. The *KCNQ1*-T600M, *KCNH2*-G294V, and *KCNE2-*V14I are therefore not classified as “functionally significant” in Table 5. Furthermore, the *KCNQ1*-T600M and *KCNH2*-G294V variants occurred in the same black victim, together with the sodium channel variant *SCN5A*-S1103Y, which has later been associated with sudden infant death in African Americans by Plant et al. [197] (see below).

As listed in Table 5, another 14 population-based cohort studies have been published since the studies by Ackerman et al. [182] and Tester and Ackerman [195]. If data on the functional significance of a mutation were not provided, the literature was searched for such data, in particular from linkage analysis and/or from expression studies. Part of the search results has been published in a previous study [210]. All of the 18 different genes tested, and therefore listed in Table 5, have previously been associated with LQTS, SQTS, BrS, or CPVT, and thus appear in Tables 1–4, except the *SCN2B* and *GJA1* genes, which encode for the sodium channel *β*_2_ subunit and the cardiac gap junction protein connexin43, respectively.

Wedekind et al. [196] performed a postmortem examination in a total of 41 SIDS cases of sudden and unexpected infant death, which occurred in 1991/1992 and 1995/1996 in the northwestern area of Germany. Apart from a number of common and rare polymorphisms, the results of screening for mutations in the LQTS genes *KCNQ1*, *KCNH2*, *SCN5A*, *KCNE1,* and *KCNE2*, associated with the LQTS types 1–3, 5, and 6, respectively, were limited to a single missense mutation in *KCNQ1* (H105L). However, the mutant channel failed to display significant electrophysiological disturbances in vitro.

Plant et al. [197] studied the prevalence of *SCN5A* variants among 133 African American SIDS cases and found homozygous and rare heterozygous *SCN5A* variants in seven cases (5%). Three cases were homozygous for S1103Y, a variant that had previously been associated with an increased risk for arrhythmia in adults [211]. Comparison to controls gave an approximately 24-fold increase in risk of SIDS with the homozygous S1103Y genotype. In vitro, the variant Y1103 channels operated normally under control conditions, but showed abnormal function when subjected to lowered intracellular pH, which may indicate a predisposition to acidosis-induced arrhythmia. Three mutations in *SCN5A* at sites other than 1103 were identified in a total of four cases—S524Y (two cases), R689H, and E1107K—and characterized as gain-of-function mutations.

The cardiac fast sodium channel is localized in caveolae, membrane microdomains involved in vesicular trafficking, whose major component in cardiomyocytes is *CAV3*-encoded caveolin-3 [212]. Vatta et al. [212] reported a novel type of LQTS (LQT9) associated with mutations in *CAV3* that result in a two- to threefold increase in late sodium current compared with wild-type caveolin-3, similar to the functional effect of LQT3-associated *SCN5A* mutations. Cronk et al. [198] investigated the prevalence of LQT9 in 134 cases of SIDS and identified three distinct *CAV3* mutations (V14L, T78M, and L79R). At functional characterization, all of these showed a significant *≈*5-fold increase in late sodium current, consistent with the LQT3-like phenotype described by Vatta et al. [212].

Between 1988 and 2004, Arnestad et al. [199] genetically analyzed 201 Norwegian cases of SIDS, screening for mutations in *KCNQ1*, *KCNH2*, *SCN5A*, *KCNE1*, *KCNE2*, *KCNJ2,* and *CAV3*, associated with LQTS types 1–3, 5–7, and 9, respectively. Mutations and rare variants in LQTS genes were identified in 26 cases. On the basis of the available functional data, 8 mutations and 7 rare variants found in 19 of 201 cases (9.5%) were considered as likely contributors to sudden death. Nine *SCN5A* mutations or rare genetic variants were found in a total of 13 cases (6.5%). Biophysical characterization of the R1193Q variant [188] and the eight other mutations or rare variants [213] revealed an increased persistent sodium current in all cases, either under control conditions or only under conditions of internal acidosis (R680H) or when expressed in the context of the common splice variant delQ1077 (delAL586-587 and V1951L). In 11 cases, nine different mutations or rare variants in the potassium channel genes* KCNQ1* (5 cases), *KCNH2* (5 cases), and *KCNE2* (one case) were found (Table 5). No mutations were found in *KCNE1* or *KCNJ2*. However, one rare *KCNQ1* variant (P448R, three cases) appeared a common, ethnic-specific polymorphism [214], whereas the mutations V279M, R885C, and S1040G in *KCNH2* (three cases in total) exhibited biophysical properties indistinguishable from wild type [215] and are probably benign variants. For the remaining five cases clear functional effects have been found [199, 215]. The functional characterization of the *KCNH2*-R273Q, *KCNH2*-R954C/K897T, and *KCNE2*-Q9E mutations revealed a loss of function of the associated current (*I*_Kr_), which would result in a long QT phenotype. The functional effect of the *KCNQ1* mutations is a loss of function (G460S) or gain of function (I274V) of the associated current (*I*_Ks_), which would result in a long QT or short QT phenotype, respectively. Arnestad et al. [199] also identified two *CAV3* variants (C72W and T78M). Since T78M had earlier been characterized as a functionally significant mutation [198], this variant was considered pathogenic, although one of the two victims with this variant also carried the functionally significant delAL586-587 mutation in* SCN5A*. With regard to C72W, no functional data are available. Of note, the C72W variant had actually been designated as a common polymorphism in the original study identifying *CAV3* as the LQT9 locus [212].

A 2004 study from the Mayo Clinic represented the first molecular autopsy of *RYR2* in cases of sudden unexplained death [216]. In the cohort of 49 cases, six distinct *RYR2 *missense mutations were found in seven cases (14%). Next, Tester et al. [200] performed a study with the aim to determine the spectrum and prevalence of *RYR2* mutations in a cohort of 134 SIDS cases. Overall, two distinct and novel *RYR2* mutations (R2267H and S4565R) were identified in two cases of SIDS that were subsequently established to be mutation negative for all known LQTS susceptibility genes. Both amino acid substitutions were absent in 400 reference alleles. Functional characterization showed that the two mutant channels were prone to display a significant gain-of-function “leaky” phenotype, especially under conditions that simulated stress during diastole. These findings are supported by the study by Mathur et al. [217], who created a knock-in mouse model of SIDS and reported that young mice with the equivalent of the gain-of-function mutation R176Q in *RYR2* [218] show an increased propensity to calcium leak-induced cardiac arrhythmias and sudden death. The nocturnal occurrence of cardiac arrhythmias in SIDS can be explained by sudden increases in sympathetic activity, for example, following hypoxia or possibly even during REM sleep [200]. 

London et al. [219] reported that the A280V mutation in the *GPD1-L* gene, which encodes the glycerol-3-phosphate dehydrogenase 1-like protein (G3PD1L), reduced inward sodium current and thus caused BrS. Given this finding, Van Norstrand et al. [201] analyzed 83 cases of sudden unexplained death, including 7 SIDS cases, and identified a *GPD1-L* mutation in a 3-month-old boy (E83K). Further analysis of a cohort of 221 SIDS cases revealed two additional mutations (I124V and R273C). All three mutations were absent in 600 reference alleles. Compared with wild type, coexpression of mutant *GPD1-L* with *SCN5A* resulted in a significantly reduced sodium current, consistent with a loss-of-function, BrS-like phenotype. London et al. [219] found a reduced cell surface expression of *SCN5A* with mutant *GPD1-L *and hypothesized that the mutation caused impaired trafficking of the cardiac sodium channel to the cell surface. Valdivia et al. [220] later provided evidence that the effect of the mutation in *GPD1-L *is through a reduced enzymatic function of G3PD1L, which regulates *SCN5A* through direct phosphorylation, whereas another study [221] emphasizes the role of a mutation-induced increase in the intracellular concentration of NADH.

In 2008, Otagiri et al. [202] reported the results of an investigation of 42 Japanese SIDS cases between 1995 and 2004, in which they studied the LQTS genes *KCNQ1*,*KCNH2,* and*SCN5A*. They identified two potassium channel mutations, one in *KCNQ1* (K598R) and one in *KCNH2* (T895M), and three mutations in *SCN5A* (F532C, G1084S and F1705S). In expression studies, the latter two *SCN5A* mutations showed hyperpolarizing shifts in inactivation (G1084S and F1705S) and a delayed recovery from inactivation (F1705S), loss-of-function features commonly seen in BrS mutations in* SCN5A*. As for *SCN5A*-F532C, Otagiri et al. [202] found no evidence indicating a functionally perturbed channel in their expression experiments, but this mutation has more recently been associated with BrS [222]. The *KCNQ1*-K598R mutation did not noticeably alter the gating of *KCNQ1* channels expressed in oocytes and was therefore considered a rare polymorphism. However, this mutation has later been identified as an LQT1-causing mutation in a clinical study [223]. Biophysical properties of *KCNH2*-T895M included a decrease in the amplitude of the steady-state current and a delay in deactivation. Since these alterations seem to exert opposite functional effects, it is difficult to predict the overall in vivo effect of this mutation, especially as the subject found in also carried the G1084S mutation in *SCN5A*.

Millat et al. [203] performed genetic screening of 32 French SIDS victims of Caucasian origin for mutations in *KCNQ1*, *KCNH2*, *SCN5A*, *KCNE1,* and *KCNE2*. They found three potassium channel mutations, one in each of the genes *KCNQ1*, *KCNH2,* and* KCNE1* (*KCNQ1*-G626S, *KCNH2*-R148W, and* KCNE1*-T20I) and none in *KCNE2*. These mutations were interpreted by the authors as a possible cause of death. With regard to the *KCNQ1* mutation, some evidence is available to support this hypothesis [224]. However, the infant with the *KCNE1* mutation, who was the subject of the aforementioned case report by Huang et al. [191], was also the carrier of a spontaneous mutation in *SCN5A* that may provide a more likely explanation for her death, given that her father was found to be a carrier of the *KCNE1*-T20I mutation with no LQTS-related phenotype [191]. The *KCNH2* defect is probably a benign polymorphism [225], as supported by the observation that the father, who also carried the mutation, was asymptomatic. Millat et al. [203] also found three mutations in *SCN5A* (Q692K, R975W, and S1333Y). The first has wild-type-like functional properties [226] and has been classified as a control variant [222], whereas the second is currently considered a rare control [222, 225]. However, *SCN5A*-S1333Y causes a defect in inactivation with the presence of a residual current, comparable to LQT3 [191].

In 2008, Ueda et al. [227] and Wu et al. [228] almost simultaneously reported that mutations in* SNTA1*, encoding the scaffolding protein *α*1-syntrophin, may lead to LQTS through a gain of function of the fast sodium channel, as in LQT3. Ueda et al. [227] found that the A390V mutation led to LQTS through increased direct S-nitrosylation of the cardiac sodium channel, resulting in a marked increase in late sodium current. The increase in late sodium current was accompanied by an increase in peak sodium current, an increase in sodium channel availability through a +6 mV shift in sodium channel inactivation, and a slower time course of sodium current decay. Wu et al. [228] studied the A257G mutation in* SNTA1* and also found an increase in late sodium current, due to an increase in peak sodium current, an increase in sodium channel availability through a –9 mV shift in sodium channel activation, and a slower time course of sodium current decay. Subsequently, Cheng et al. [204] investigated the prevalence and functional properties of *SNTA1* mutations in a cohort of 292 SIDS cases. Six mutations were found in eight cases, with one particular mutation (P56S) identified in three cases, and were absent in 800 reference alleles. In vitro, a significant increase in peak and late sodium current was observed for S287R, T372M, and G460S, which was reversed by a neuronal nitric oxide synthase inhibitor. The other three variants (G54R, P56S, and T262P) showed functionally insignificant changes in the sodium current.

Tan et al. [205] screened the Mayo Clinic cohort of 292 unrelated SIDS victims for mutations in the four sodium channel *β* subunit genes *SCN1B* to *SCN4B* and identified a total of three mutations in two of these genes (*SCN3B*-V36M, *SCN3B*-V54G, and *SCN4B*-S206L). The two *SCN3B* mutations are both localized in the extracellular loop of the *β* subunit, which is important for *β*3 membrane trafficking. Compared with wild-type channels, V36M channels showed both loss-of-function and gain-of-function phenotypes in expression studies, that is, decreased peak *I*_Na_ and increased late *I*_Na_ (at least when normalized to peak *I*_Na_), respectively, whereas V54G was purely a loss of function. *SCN4B*-S206L is associated with an increase in late sodium current, as demonstrated through the adenoviral transduction of adult rat ventricular myocytes [205]. 

Tester et al. [206] performed a mutational analysis of *KCNJ8*, which encodes the *α* subunit of the ATP-sensitive potassium channel, on genomic DNA obtained from the SIDS victims of the Mayo Clinic cohort. Two distinct and novel *KCNJ8* mutations were identified (E332del and V346I). Both cases were negative for mutations in established channelopathic genes. In vitro, both mutations resulted in a significant decrease in the associated ATP-sensitive potassium current (*I*_K,ATP_). Loss of function of the *KCNJ8*-encoded cardiac *K*_ATP_ channel may result in a long QT phenotype, predisposing to SIDS, but an isolated pro-arrhythmic mechanism remains speculative since *KCNJ8* is expressed in multiple tissues, including vascular and neuronal tissue [206].

Na_v_ *β*1B, encoded by *SCN1Bb*, is a splicing variant of the *β*_1_ subunit of the *I*_Na_ channel [229]. Mutations in *SCN1Bb* have been associated with BrS (Table 2). Hu et al. [207] have assessed the Mayo Clinic SIDS cohort for mutations in *SCN1Bb* and found a functional rare variant in a single SIDS victim who had died at the age of 4 months. In coexpression studies with wild-type *SCN5A* or *KCND3*, the R214Q variant had a loss-of-function effect on *I*_Na_ as well as a gain-of-function effect on *I*_to_, in line with the reported association with BrS.

Van Norstrand et al. [208] detected two novel missense mutations (E42K and S272P) in *GJA1*, which encodes the gap junction channel protein connexin43 (Cx43), in the Mayo Clinic cohort of 292 SIDS cases. Functional studies were performed using dual whole cell patch clamp and revealed a strongly reduced gap junctional conductance for E42K compared to wild type. Such strongly reduced intercellular coupling may result in lethal ventricular arrhythmias, as demonstrated in several conditional Cx43 knockout models [230].

When screening the Mayo Clinic cohort for mutations in *KCND3*, which encodes the *α* subunit of the *I*_to_ channel, Giudicessi et al. [209] found a rare missense mutation in a single SIDS victim who had died at the age of 2 months. However, when the S530P mutation was heterologously expressed in HEK293 cells, the electrophysiological phenotype did not differ from wild type. 

## 5. Prevalence of Cardiac Ion Channelopathies in SIDS

**Figure 7:**
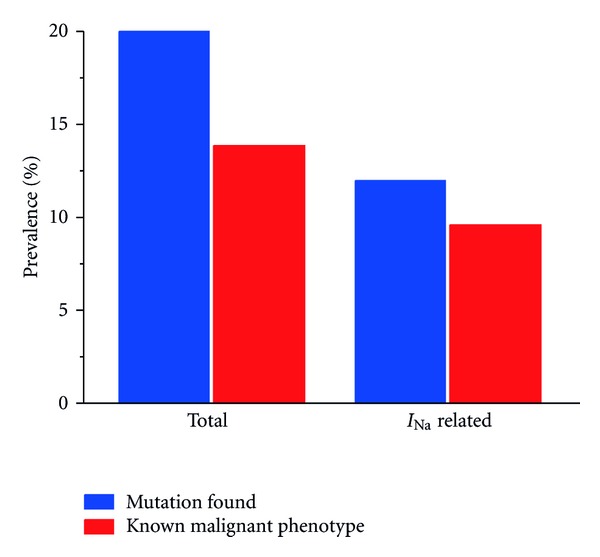
Prevalence of cardiac ion channelopathies in SIDS victims, based on mutations found in population-based cohort studies (Tables 5 and 6). Left: overall prevalence (blue bar) and prevalence of mutations with a known malignant phenotype (red bar). Right: prevalence of fast sodium current (*I*_Na_) related mutations (blue bar) and those of a known malignant phenotype (red bar).

**Figure 8:**
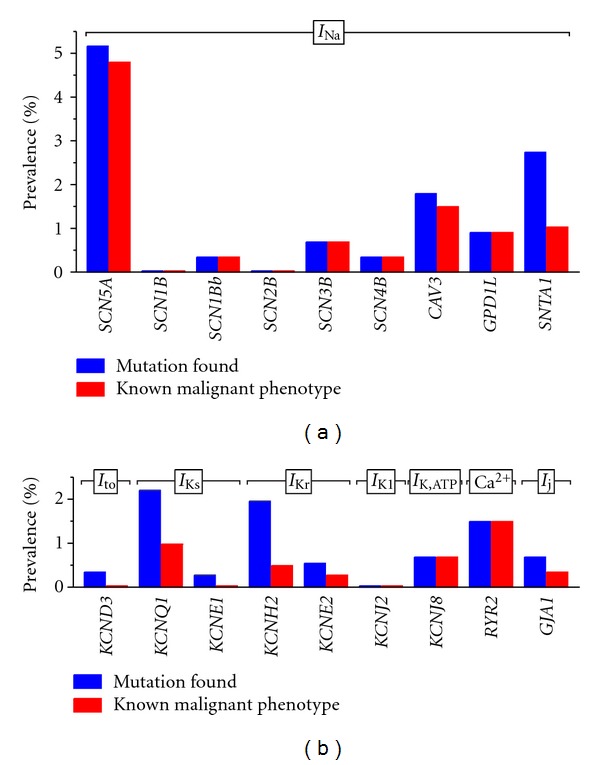
Prevalence of cardiac ion channelopathies in SIDS victims, based on data from population-based cohort studies (Tables 5 and 6). Blue bars indicate the prevalence of mutations and red bars that of mutations with a known malignant phenotype. (a) Mutations in genes affecting the fast sodium current *I*_Na_. (b) Mutations in genes affecting the transient outward potassium current *I*_to_ (*KCND3*), the slow delayed rectifier potassium current *I*_Ks_(*KCNQ1* and *KCNE1*), the rapid delayed rectifier potassium current *I*_Kr_ (*KCNH2* and *KCNE2*), the inward rectifier potassium current *I*_K1_ (*KCNJ2*), the ATP-sensitive potassium current *I*_K,ATP_ (*KCNJ8*), the RyR2 calcium release channel (*RYR2*), and the gap junctional current *I*_j_ (*GJA1*).

From the data in Table 5, one can compute the prevalence of mutations in each of the genes tested thus far. Mutations in the sodium channel gene *SCN5A*, for example, have been assessed in the studies by Ackerman et al. [182], Wedekind et al. [196], Plant et al. [197], Arnestad et al. [199], Otagiri et al. [202], and Millat et al. [203], yielding a total of 542 SIDS cases, in which 28 mutations in *SCN5A* were detected (5.2%), almost all with functional significance (26, or 4.8%). Similarly, numbers for the other 17 genes can be obtained. All results appear in Table 6 and are illustrated in Figures 7 and 8. It should be noted that, when compiling Tables 5 and 6, it has been ignored that a few SIDS victims carried two ion channel-related mutations at the same time and thus they appear in these tables twice. This holds, as mentioned before, for an infant in the study by Tester and Ackerman [195] with mutations in both *KCNQ1* and *KCNH2*, for an infant in the study by Arnestad et al. [199] with *CAV3*-T78M and *SCN5A*-delAL586-587 mutations, and for an infant in the study by Millat et al. [203] with *KCNE1*-T20I and *SCN5A*-S1333Y mutations. Furthermore, it has been ignored that there are differences in the populations between studies, which are mostly Northern American, but also African American [197], French [203], German [196], Norwegian [199], and Japanese [202].

According to the data in Table 6, 20.2% of the SIDS victims carried a mutation in any of the 18 cardiac ion channel-related genes studied (Figure 7, leftmost bar). In the majority of cases (13.9%), there is evidence for malignancy of these mutations, as detailed in Section 4.2, with functional effects related to LQTS types 1–3, 5, 6, 9, 10, and 12, BrS types 1, 2, and 7, and SQTS type 2 and CPVT type 1. In the remaining cases (6.3%), there are either no data on the functional effects of the mutation or no functionally significant effects have been observed, for example, in expression studies. In the latter case, there may still be latent functional defects that are not evident under the conditions of the functional test. For example, there may be defects that only show their malignancy in the case of acidosis or in the context of a specific splice variant, as for some of the SIDS-associated *SCN5A* variants studied by Wang et al. [213] (see Section 4.2). Another example is the SIDS associated *KCNQ1*-K598R mutation, which seemed benign when expressed in oocytes [202], but was later identified as an LQT1-causing mutation in a clinical study [223]. Similarly, the D1275N mutation in *SCN5A* showed a clear clinical phenotype and an impaired sodium current in a mouse model, but did not generate major changes in sodium channel function in heterologous expression studies [231].

Another factor that may turn apparently benign mutations into malignant ones is high temperature. The malignant properties of a mutation may become apparent or more severe with an increase in temperature, as demonstrated for several sodium channel mutations associated with BrS, for example, in the studies by Dumaine et al. [232] and Keller et al. [233], explaining fever-induced arrhythmias observed in BrS patients. More recently, it has become clear that fever may also induce life-threatening arrhythmias in the carriers of an LQT2 mutation [234, 235]. The aforementioned case report by Chockalingam et al. [194] provides evidence that arrhythmogenic events triggered by fever, in this case vaccination related, may also play a role in the etiology of SIDS. A second case has recently been presented, as part of a study on the effects of loss-of-function sodium channelopathies in children [236]. In addition, Kanter et al. [237] reported a case of rapid ventricular tachycardia in a 5-month-old boy on the day of receiving his 6-month immunizations.

If we assume that at least some of the mutations for which functional data are lacking are of a malignant phenotype, the prevalence of malignant cardiac ion channel-related mutations among SIDS victims may actually be close to 20%. It may even be higher, given that conventional genetic analysis may fail to uncover severe mutations [238–242] and that the determination of associations between SIDS and mutations in ion channel-related genes is still an emerging field, with several associations that have only recently been reported [206–208] and others that remain to be assessed, for example, a possible association between SIDS and mutations in the L-type calcium channel-related genes *CACNA1C*, *CACNB2b,* and *CACNA2D1*, which have been associated with LQTS, BrS, and SQTS (Table 1–3). Of note, the prevalence of LQTS, and BrS-associated mutations among SIDS victims is much higher than the estimated prevalence of 1 : 2,000 for LQTS and 1 : 2,000 to 1 : 5,000 for BrS in the general population [97, 127].

Furthermore, we can conclude that mutations in *I*_Na_ channel-related genes are the most malignant ones. Mutations in the *α* subunit encoding gene, *SCN5A*, are found in 5.2% of the SIDS victims (Table 6, Figure 8(a)), in accordance with the severe symptoms in children with LQT3 [118] and the observation in adults that LQT3 has the most severe clinical events [117] and that these events tend to occur during sleep [243], as in SIDS. In total, more than half of the reported mutations are related to the *I*_Na_ channel (Figure 7). In addition to the mutations in *SCN5A*, there are mutations in the *β* subunit encoding genes* SCN1Bb*,* SCN3B,* and *SCN4B* (1.4%) and in “regulatory genes” (*CAV3*, *GPD1-L, *and *SNTA1*; 5.4%), adding up to a prevalence of 12.0% for mutations affecting *I*_Na_, with functional evidence available for 9.6% (Table 6, Figures 7 and 8(a)). Similar percentages for the “cardiac sodium Nav1.5 channelsome” have been reported in an abstract form by Van Norstrand et al. [244]. With a total prevalence of 6.0%, with functional evidence in the case of 2.4%, mutations in potassium channel genes (*KCND3*, *KCNQ1*, *KCNE1*, *KCNH2*, *KCNE2*, *KCNJ2,* and *KCNJ8*) seem less frequent and less malignant (Figure 8(b)).

It should be noted that mutations in ion channel-related genes as putative causes of death are not limited to cases of SIDS, but have also been reported in cohorts of sudden unexplained death cases in which the victims were >1 year of age [113, 245–247]. Also, there are reports, for example, by Cuneo et al. [248] and Miller et al. [249], that mutations in ion channel-related genes may lead to cardiac arrhythmias and death in utero. 

## 6. Conclusion

In addition to clinical associations such as prone sleeping and exposure to cigarette smoke, several genetic factors have been identified with regard to SIDS, including polymorphisms in genes related to the immune system and the autonomic nervous system. With large prospective ECG studies, several “proof-of-concept” cases, a series of population-based cohort studies, and the ability to assess the functional effects of a mutation in vitro, the role of cardiac ion channel-related mutations as a genetic contributor to SIDS is currently the most well established. Combining data from population-based cohort studies, it can be concluded that one out of five SIDS victims carries a mutation in a cardiac ion channel-related gene and that the majority of these mutations are of a known malignant phenotype.

## 7. Further Reading

The intriguing issue of the role of mutations in ion channel-related genes in cases of sudden unexplained deaths has been addressed in a number of recent reviews. For further reading the reader is referred to the reviews by Tester and Ackerman [250], Van Norstrand and Ackerman [251, 252], Tfelt-Hansen et al. [253], Cerrone and Priori [254], Chopra and Knollmann [255], Insolia et al. [256], Opdal and Rognum [257], Martin et al. [258] and Perrin and Gollob [259].

## Figures and Tables

**Table 1:** Genes linked to long QT syndrome.

Type	OMIM	Gene	Protein	Functional role in cardiomyocytes	Effect of mutation
			Autosomal dominant inheritance		

LQT1	#192500	*KCNQ1 (KVLQT1) *	K_v_7.1 (K_v_LQT1)	*α* subunit of *I*_Ks_ channel	Loss of function of *I*_Ks_
LQT2	#613688	*KCNH2 (HERG) *	K_v_11.1	*α* subunit of *I*_Kr_ channel	Loss of function of *I*_Kr_
LQT3	#603830	*SCN5A *	Na_v_1.5	*α* subunit of *I*_Na_ channel	Gain of function of *I*_Na_
LQT4	#600919	*ANK2 *	Ankyrin-B	Anchoring protein	Loss of function of multiple ion channels and transporters
LQT5	#613695	*KCNE1 *	KCNE1 (minK)	*β* subunit of *I*_Ks_ channel	Loss of function of *I*_Ks_
LQT6	#613693	*KCNE2 *	KCNE2 (MiRP1)	*β* subunit of *I*_Kr_ channel	Loss of function of *I*_Kr_
LQT7^‡^	#170390	*KCNJ2 *	K_ir_2.1	*α* subunit of *I*_K1_ channel	Loss of function of *I*_K1_
LQT8^†^	#601005	*CACNA1C *	Ca_v_1.2	*α*_ 1C_ subunit of *I*_Ca,L_ channel	Gain of function of *I*_Ca,L_
LQT9	#611818	*CAV3 *	Caveolin-3	Caveolar coating	Gain of function of *I*_Na_
LQT10	#611819	*SCN4B *	Na_v_ *β*4	*β* subunit of *I*_Na_ channel	Gain of function of *I*_Na_
LQT11	#611820	*AKAP9 *	Yotiao	Anchoring protein for PKA regulatory subunit	Loss of function of *I*_Ks_
LQT12	#612955	*SNTA1 *	*α*_ 1_-syntrophin	Scaffolding protein	Gain of function of *I*_Na_
LQT13	#613485	*KCNJ5 *	K_ir_3.4	*α* subunit of *I*_K,ACh_ channel	Loss of function of *I*_K,ACh_

			Autosomal recessive inheritance		

JLN1	#220400	*KCNQ1 (KVLQT1) *	K_v_7.1 (K_v_LQT1)	*α* subunit of *I*_Ks_ channel	Loss of function of *I*_Ks_
JLN2	#612347	*KCNE1 *	KCNE1 (minK)	*β* subunit of *I*_Ks_ channel	Loss of function of *I*_Ks_

OMIM: Online Mendelian Inheritance in Man compendium of human genes and genetic phenotypes; LQT1– LQT13: long QT syndrome types 1–13; JLN1 and JLN2: Jervell and Lange-Nielsen syndrome types 1 and 2; *I*_Ca,L_: L-type calcium current; *I*_K,ACh_: acetylcholine-sensitive potassium current; *I*_K1_: inward rectifier potassium current; *I*_Kr_: rapid delayed rectifier potassium current; *I*_Ks_: slow delayed rectifier potassium current; *I*_Na_: fast sodium current.

^‡^Also known as Andersen's syndrome or Andersen-Tawil syndrome

^†^Also known as Timothy syndrome.

**Table 2:** Genes linked to Brugada syndrome.

Type	OMIM	Gene	Protein	Functional role in cardiomyocytes	Effect of mutation
			Autosomal dominant inheritance		

BrS1	#601144	*SCN5A *	Na_v_1.5	*α* subunit of *I*_Na_ channel	Loss of function of *I*_Na_
BrS2	#611777	*GPD1-L *	G3PD1L	Not fully established	Loss of function of *I*_Na_
BrS3	#611875	*CACNA1C *	Ca_v_1.2	*α*_1C_ subunit of *I*_Ca,L_ channel	Loss of function of *I*_Ca,L_
BrS4	#611876	*CACNB2b *	Ca_v_ *β*2b	*β*_2b_ subunit of *I*_Ca,L_ channel	Loss of function of *I*_Ca,L_
BrS5	#612838	*SCN1B *	Na_v_ *β*1	*β* subunit of *I*_Na_ channel	Loss of function of *I*_Na_
BrS6	#613119	*KCNE3 *	KCNE3 (MiRP2)	*β* subunit of voltage-dependent K^+^ channels	Gain of function of *I*_to_
BrS7	#613120	*SCN3B *	Na_v_ *β*3	*β* subunit of *I*_Na_ channel	Loss of function of *I*_Na_
NC	#613123	*HCN4 *	HCN4	*α* subunit of *I*_f_ channel	Gain of function of *I*_f_
NC	—	*CACNA2D1 *	Ca_v_ *α*_2_ *δ*-1	*α*_ 2_ *δ* subunit of *I*_Ca,L_ channel	Loss of function of *I*_Ca,L_
NC	—	*MOG1 *	MOG1	Regulates trafficking of Na_v_1.5 to the membrane	Loss of function of *I*_Na_
NC	—	*KCND3 *	K_v_4.3	*α* subunit of *I*_to_ channel	Gain of function of *I*_to_
NC	—	*KCNE1L (KCNE5) *	KCNE1L	*β* subunit of voltage-dependent K^+^ channels	Gain of function of *I*_to_
NC	—	*KCNJ8 *	K_ir_6.1	*α* subunit of *I*_K,ATP_ channel	Gain of function of *I*_K,ATP_
NC	—	*SCN1Bb *	Na_v_ *β*1B	*β* subunit of *I*_Na_ channel	Loss of function of *I*_Na_ and gain of function of *I*_to_

OMIM: Online Mendelian Inheritance in Man compendium of human genes and genetic phenotypes; BrS1–BrS7: Brugada syndrome types 1–7; NC: no consensus; *I*_Ca,L_: L-type calcium current; *I*_f_: hyperpolarization-activated current; *I*_K,ATP_: ATP-sensitive potassium current; *I*_Na_: fast sodium current; *I*_to_: transient outward potassium current.

**Table 3:** Genes linked to short QT syndrome.

Type	OMIM	Gene	Protein	Functional role in cardiomyocytes	Effect of mutation
			Autosomal dominant inheritance		

SQT1	#609620	*KCNH2 (HERG) *	K_v_11.1	*α* subunit of *I*_Kr_ channel	Gain of function of *I*_Kr_
SQT2	#609621	*KCNQ1 (KVLQT1) *	K_v_7.1 (K_v_LQT1)	*α* subunit of *I*_Ks_ channel	Gain of function of *I*_Ks_
SQT3	#609622	*KCNJ2 *	K_ir_2.1	*α* subunit of *I*_K1_ channel	Gain of function of *I*_K1_
SQT4	—	*CACNA1C *	Ca_v_1.2	*α*_1C_ subunit of *I*_Ca,L_ channel	Loss of function of *I*_Ca,L_
SQT5	—	*CACNB2b *	Ca_v_ *β*2b	*β*_2b_ subunit of *I*_Ca,L_ channel	Loss of function of *I*_Ca,L_
SQT6	—	*CACNA2D1 *	Ca_v_ *α*_2_ *δ*-1	*α*_ 2_ *δ* subunit of *I*_Ca,L_ channel	Loss of function of *I*_Ca,L_

OMIM: Online Mendelian Inheritance in Man compendium of human genes and genetic phenotypes; SQT1–SQT6: short QT syndrome types 1–6; *I*_Ca, L_: L-type calcium current; *I*_K1_: inward rectifier potassium current; *I*_Kr_: rapid delayed rectifier potassium current; *I*_Ks_: slow delayed rectifier potassium current.

**Table 4:** Genes linked to catecholaminergic polymorphic ventricular tachycardia.

Type	OMIM	Gene	Protein	Functional role in cardiomyocytes	Effect of mutation
			Autosomal dominant inheritance		

CPVT1	#604772	*RYR2 *	RyR2	Ryanodine receptor in SR membrane	Calcium leak from SR

			Autosomal recessive inheritance		

CPVT2	#611938	*CASQ2 *	Calsequestrin-2	Calcium buffering in SR	Disrupted SR calcium buffering capacity
NC	—	*TRDN *	Triadin	Links calsequestrin-2 and RyR2	Impaired SR calcium release

OMIM: Online Mendelian Inheritance in Man compendium of human genes and genetic phenotypes; CPVT1 and CPVT2: catecholaminergic polymorphic ventricular tachycardia types 1 and 2; NC: no consensus; SR: sarcoplasmic reticulum.

**Table 5:** Mutations or rare variants in cardiac ion channel-related genes in SIDS cohorts.

Study^‡^	Size of cohort	Genes tested	Reported mutations or rare variants^†^
Ackerman et al. [182]	93	*SCN5A *	**A997S**, **R1826H**

Tester and Ackerman [195]	93	*KCNQ1 *	T600M
			*KCNH2 *
			*KCNE1 *
			*KCNE2 *

Wedekind et al. [196]	41	*SCN5A *	—
			*KCNQ1 *
			*KCNH2 *
			*KCNE1 *
			*KCNE2 *

Plant et al. [197]	133	*SCN5A *	**S524Y** (2 cases), **R689H**, **homozygous S1103Y** (3 cases), **E1107K**

Cronk et al. [198]	134	*CAV3 *	**V14L**, **T78M**, **L79R**

Arnestad et al. [199]	201	*SCN5A *	**S216L**, **delAL586-587**, **R680H**, **R1193Q** (2 cases), **T1304M**, **F1486L**, **V1951L**, **F2004L** (3 cases), **P2006A** (2 cases)
			*KCNQ1 *
			*KCNH2 *
			*KCNE1 *
			*KCNE2 *
			*KCNJ2 *
			*CAV3 *

Tester et al. [200]	134	*RYR2 *	**R2267H**, **S4565R**

Van Norstrand et al. [201]	221	*GPD1-L *	**I124V**, **R273C**

Otagiri et al. [202]	42	*SCN5A *	**F532C**, **G1084S**, **F1705S**
			*KCNQ1 *
			*KCNH2 *

Millat et al. [203]	32	*SCN5A *	Q692K, R975W, **S1333Y**
			*KCNQ1 *
			*KCNH2 *
			*KCNE1 *
			*KCNE2 *

Cheng et al. [204]	292	*SNTA1 *	G54R, P56S (3 cases), T262P, **S287R**, **T372M**, **G460S**

Tan et al. [205]	292	*SCN1B *	—
			*SCN2B *
			*SCN3B *
			*SCN4B *

Tester et al. [206]	292	*KCNJ8 *	**E332del**, **V346I**

Hu et al. [207]	292	*SCN1Bb *	**R214Q**

Van Norstrand et al. [208]	292	*GJA1 *	**E42K**, S272P

Giudicessi et al. [209]	292	*KCND3 *	S530P

^‡^Studies in order of (online) publication.

^†^Functionally significant mutations or rare variants listed in bold.

**Table 6:** Prevalence of cardiac ion channel-related mutations in SIDS cohorts^‡^.

		Number of SIDS cases	
Gene	Total	with mutation	Functionally significant
*I*_Na_ related genes			
*SCN5A *	542	28 (5.2%)	26 (4.8%)
*SCN1B *	292	0	0
*SCN1Bb *	292	1 (0.3%)	1 (0.3%)
*SCN2B *	292	0	0
*SCN3B *	292	2 (0.7%)	2 (0.7%)
*SCN4B *	292	1 (0.3%)	1 (0.3%)
*CAV3 *	335	6 (1.8%)	5 (1.5%)
*GPD1-L *	221	2 (0.9%)	2 (0.9%)
*SNTA1 *	292	8 (2.7%)	3 (1.0%)
*I*_to_ related genes			
*KCND3 *	292	1 (0.3%)	0
*I*_Ks_ related genes			
*KCNQ1 *	409	9 (2.2%)	4 (1.0%)
*KCNE1 *	367	1 (0.3%)	0
*I*_Kr_ related genes			
*KCNH2 *	409	8 (2.0%)	2 (0.5%)
*KCNE2 *	367	2 (0.5%)	1 (0.3%)
*I*_K1_ related genes			
*KCNJ2 *	201	0	0
*I*_K,ATP_ related genes			
*KCNJ8 *	292	2 (0.7%)	2 (0.7%)
Intracellular calcium cycling related genes			
*RYR2 *	134	2 (1.5%)	2 (1.5%)
Gap junctional current related genes			
*GJA1 *	292	2 (0.7%)	1 (0.3%)

*I*_K,ATP_: ATP-sensitive potassium current; *I*_K1_: inward rectifier potassium current; *I*_Kr_: rapid delayed rectifier potassium current; *I*_Ks_: slow delayed rectifier potassium current; *I*_Na_: fast sodium current; *I*_to_: transient outward potassium current.

^‡^Based on data listed in Table 5.
